# Novel Insights Into Monogenic Obesity Syndrome Due to *INPP5E* Gene Variant: A Case Report of a Female Patient

**DOI:** 10.3389/fendo.2021.581134

**Published:** 2021-06-15

**Authors:** Ana Drole Torkar, Magdalena Avbelj Stefanija, Sara Bertok, Katarina Trebušak Podkrajšek, Maruša Debeljak, Branislava Stirn Kranjc, Tadej Battelino, Primož Kotnik

**Affiliations:** ^1^ University Children’s Hospital, Department of Endocrinology, Diabetes and Metabolism, Ljubljana University Medical Centre, Ljubljana, Slovenia; ^2^ University Children’s Hospital, Unit of Special Laboratory Diagnostics, Ljubljana University Medical Centre, Ljubljana, Slovenia; ^3^ Faculty of Medicine, University of Ljubljana, Ljubljana, Slovenia; ^4^ University Eye Hospital, Ljubljana University Medical Centre, Ljubljana, Slovenia

**Keywords:** MORMS, *INPP5E* gene, monogenic obesity, retinal dystrophy, case report

## Abstract

A Caucasian girl with consanguineous parents presented with early severe obesity and retinal dystrophy. A novel, homozygous gene truncating variant (c.1897C>T) in the *INPP5E* gene confirmed the diagnosis of MORMS (OMIM #610156). A novel clinical finding in the presented syndrome is progressive cone-rod type retinal dystrophy diagnosed at the age of four months that progressed in the 1^st^ decade of life. Severe obesity, insulin resistance with hyperinsulinism, and impaired glucose tolerance developed alongside other components of the metabolic syndrome - dyslipidemia, arterial hypertension, and obstructive hypopnea in sleep. At the age of 14 years, primary amenorrhea persists. The patient is managed by regular nutritional advice, metformin, antihypertensive medication, and non-invasive respiratory support during sleep. Differential diagnosis of this rare entity is discussed in extend.

## Introduction

Monogenic obesity is rare, it presents, early and is severe, with minimal environmental influence on the phenotype (1). Characterization of genes involved in the development of monogenic obesity also contributed to the unraveling of mechanisms leading to polygenetic/common obesity and could contribute to the development of novel targeted therapeutic interventions (2).

Gene encoding inositol polyphosphate-5-phosphatase E (*INPP5E*) is located on chromosome 9q34. It is widely expressed in humans, especially in the brain (3, 4). INPP5E belongs to the 5-ptase family of enzymes and is involved in regulating diverse cellular processes, namely synaptic vesicle recycling, insulin signaling, and embryonic development (5–7). Loss of INPP5E function leads to decreased cilia stability and ciliopathy (5–9) as it plays an essential role in the primary cilium by controlling ciliary growth factor and phosphoinositide 3-kinase (PI3K) signaling and stability (2, 3, 5, 6, 10). Primary cilia play a critical role in the development and functioning of several cell types, including retinal photoreceptors, neurons, kidney tubules, and bile ducts (11, 12). Pathogenic variants in the INPP5E gene are linked to two partly overlapping but clinically distinct autosomal recessive ciliopathies: Joubert syndrome 1 (JBTS1; OMIM #213300) characterized by the malformation of the brainstem and agenesis or hypoplasia of the cerebellar vermis that causes abnormal respiratory pattern, nystagmus, hypotonia, ataxia, and developmental delay (6, 13); and a syndrome of static moderate mental disability, truncal obesity, congenital non-progressive retinal dystrophy, and micropenis in males (MORMS; OMIM #610156) (13).

In the case of MORMS, a nonsense mutation is located in the C-terminal domain of the gene, resulting in a truncated protein, affecting ciliary localization and stabilization of ciliary structures (5, 14).

MORMS has been, to our knowledge, so far described in a single complex consanguineous Pakistani kindred (13). We present a novel case of MORM syndrome in a Caucasian girl with consanguineous parents, including several novel clinical features.

## Case Presentation

### Family Medical History

**Figure 1:**
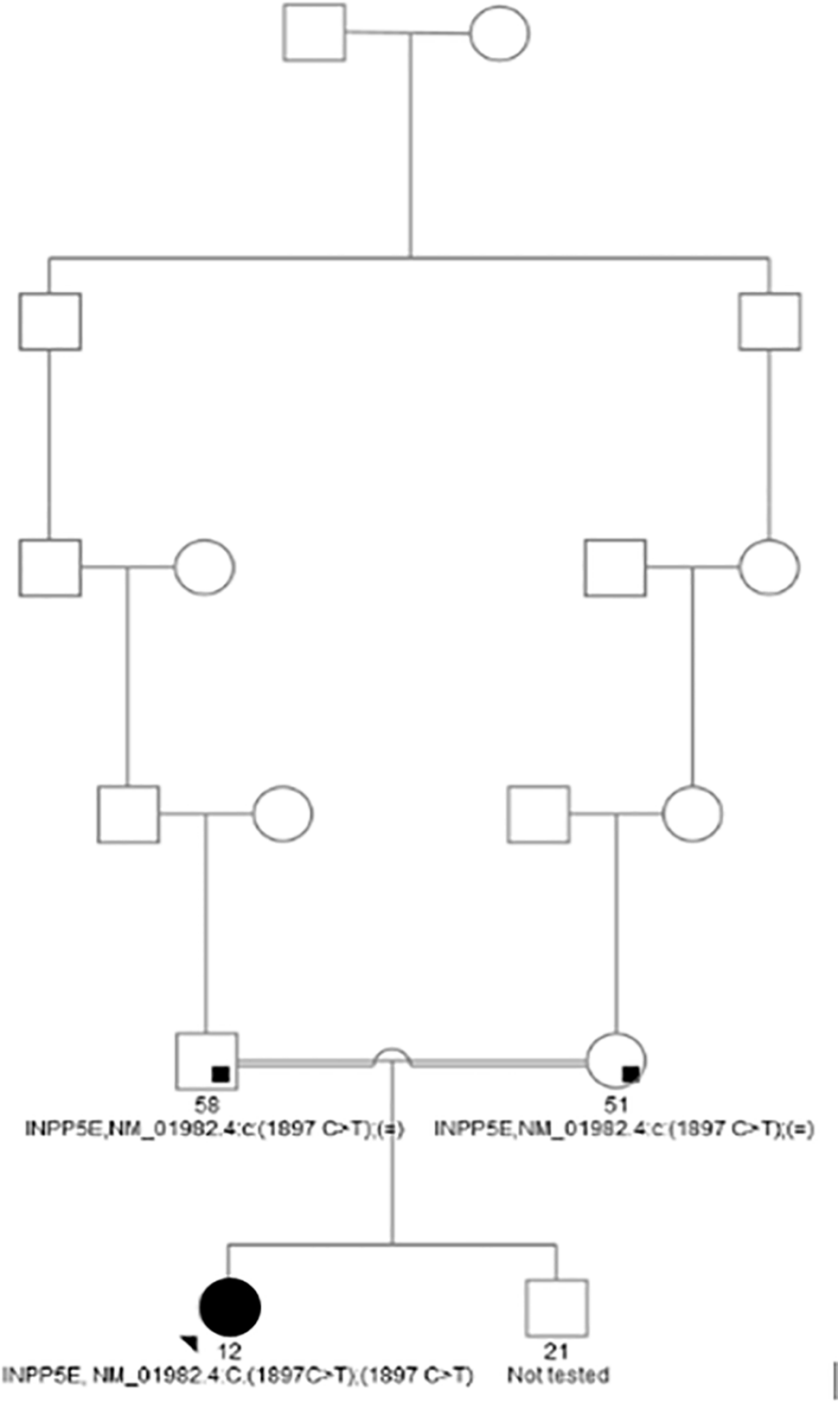
Family tree of the proband.

Girl’s maternal and paternal grand grandfathers were brothers (**Figure 1**). The father has arterial hypertension, and the mother is overweight and had dermatitis and cholelithiasis. The girl has an older brother, who is also overweight, without visual impairment, but has not been available for further clinical or genetic evaluations. There is no data on intellectual disability, extreme obesity, or impaired vision in the extended family.

### Clinical Observations and Investigations

The girl was born at term following mother’s uneventful second pregnancy, with a birth weight of 4.23 kg (SDS-W 1.8) and a birth length of 51 cm (SDS-L 1.5). There were no complications in the neonatal period.

### Retinal Dystrophy of Cone-Rod Type

**Figure 2:**
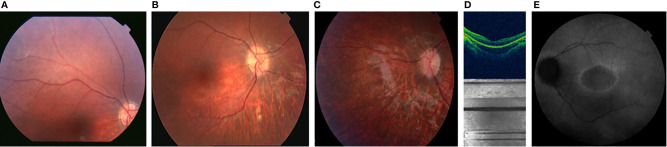
Color fundus photographs [**(A)** at the age of 2 yrs.], [**(B)** at the age of 6 yrs. [**(C)** at the age of 7 yrs.] showing the progression of the optic disc pallor with retinal arteriolar narrowing and pigment mottling; OCT image [**(D)** at the age of 7 years, showing thinner retina predominantly the outer retinal layer with irregularities] AF [**(E)** at the age of 10 years, showing hyper-fluorescent ring around the macula with irregular fluorescence of the posterior pole].

The first presenting sign of MORMS was horizontal nystagmus of low frequency and amplitude at the age of four months. At the age of two years, visual acuity was normal for age, but decreased to moderate myopic astigmatism by the age of ten years. The diagnosis of retinal dystrophy was confirmed at the age of five years, three years after some discrete pigmentary changes of the retina were first noticed. Retinal appearance showed progressive retinal dystrophy with optic disc pallor, attenuated retinal vessels, loss of foveal reflex and retinal pigment mottling in the posterior pole, and the retinal periphery. Electrophysiological examination (scotopic photopic electroretinography) confirmed cone-rod type retinal dystrophy. An uneven foveal reflex with a hyper-fluorescent ring around the macula was seen on fundus auto-fluorescence retina angiograph (HRA, Spectralis Heidelberg). 3D Optic Coherence Topography (Topcon 1000) analysis determined reduced retinal thickness with irregularities, predominantly of the outer retinal layer (**Figure 2**). Orthophoria, reduced visual acuity, and color vision with concentrically narrowed visual fields became evident. No signs of cataract developed in the 1^st^ decade.

### Obesity and Metabolic Syndrome Evaluation

Development of obesity, the second hallmark of MORMS, was noticed when a substantial weight gain from the age of six months (7.5 kg, SDS-W 0.2) to 12 months (14 kg, SDS-W 3.5) and 12 to 18 months (20.5 kg, SDS-W 5.2) was determined and pronounced food-seeking behavior was described. She was first presented to the pediatric endocrinologist at the age of two years. At the first visit, marked hyperinsulinism with normoglycemia and dyslipidemia were already determined (**Table 1**). Therapy with metformin was started at the age of five years. Obesity and severe hyperphagia progressed. Impaired glucose tolerance was determined at the age of ten years (**Table 1**).

**Table 1:** Timeline of clinical findings, Diagnostic tests, and Interventions.

Patient age	Summaries from initial and Follow-up Visits	Diagnostic Testing		Interventions
		Biochemical results	Radiological and other results	
10 months	Obesity: weight 11.5 kg (+2.4 SD) Concomitant alternant strabismus Horizontal nystagmus from the age of 6 months on	Psychological evaluation (BSID-II): -mental development index 100; motor development index 84; appropriate for 8-months	Brain MRI evaluation(performed at 6 months): unmyelinated deep white metter frontoparietally in T2 sequences Hearing test: at 20 dB (normal for age) Somatosensory evoked potentials – N.medianus prolonged latencies of cortical conduction and response	Diet advice
2.5 years	Obesity: weight 33.25 kg (+5.56 SD), BMI-SDS + 5.36 No acanthosis nigricans Pseudotelarche Striae on the legs Horizontal nystagmus; visual acuity appropriate for age, pigmentary changes of the retina, narrow arterioles with mild optic disc pallor	Glucose tolerance: fasting glucose 4.5 mmol/l; 120 ' glucose 6.7 mmol/l, 120' IRI 135 mIU/L, Homa-IR 1.86, HbA1c 5.3 % Dislipidemia: Total cholesterol 5.4/LDL 3.7, HDL 1/TGL 1.4 mmol/l AST 1.7, ALT 0.61, GGT 0.35, CK 0.66 µkat/L	CMCRF normal Blood pressure: 85/61-95/72-85/52 mmHg (0.92 SDS score) US of abdomen: moderate steatosis hepatis, size of liver and spleen on the upper limit of normal for age ECHO of the heart: normal	Diet advice with 1200 kcal/day limitation
5 years	Obesity: weight 63.2 kg (+ 6.53 SD), BMI- SDS + 6.03, waist circumference-SDS + 6.16 Rejected medically supervised weight-loss program Striae No acanthosis nigricans Pseudotelarche Early signs of retinal dystrophy of cone-rod type, visual acuity right eye 0.5/ left eye 0.1 No sleep-apnea reports	Glucose tolerance: fasting glucose 4.9 mmol/l; 120 ' glucose 6.6 mmol/l, 120' IRI 155 mIU/L, Homa-IR 2.61, HbA1c 5.2 % Dislipidemia: Total cholesterol 5.5/LDL 3.8, HDL 0.9/TGL 1.8 mmol/l, Lp(a) < 93.1 mg/L	Bone-age + 2.45, Hypertension: 139 (5.81 SDS score)/65 (2.87 SDS) mmHg The US of the abdomen without pathological findings ECHO of the heart: the left ventricle size on the upper normal limit, with no signs of dilatative cardiomyopathy Hearing tests: normal No skeletal anomalies of the extremities on radiological examination	Diet advice Metformin 250 mg/12 h
6 years	Obesity: weight 73.25 kg (+ 5.54 SD), BMI- SDS + 4.77, waist circumference-SDS + 4.82 Acanthosis nigricans Visual acuity of 0.15 Rejected guided medical weight reduction program	Glucose tolerance: Fasting glucose 4.6 mmol/l, HOMA-IR 6.48 Dyslipidemia; Cholesterol 5.0/LDL 3.3/HDL 0.9 /TGL 1.9 mmol/L	Hipertension 134 (SDS 2.74) /71 (1.67 SDS) mmHg	Diet advice Metformin 500 mg/12 h
10.5 years	Obesity: weight 115.25 kg (+ 4.58 SD), BMI- SDS + 3.97 Pubertal Tanner stage: A2, P2, pseudotelarche 10 % of best-corrected visual acuity	Glucose tolerance: Fasting glucose 5.2 mmol/l, Glucose on 120 ' OGTT 8.8 mmol/l; IRI on 120' 792; HOMA-IR 12.77, HbA1c 5.5% TSH 2.86 mE/L, pT4 16.9 pmol/L, ACTH 3.33 pmol/L, Cortisol 326 nmol/L, IGF-1 90 µg/L; IGFBP-3 2.75 mg/L	Bone age + 3 SD Hypertension 129 (1.83 SDS score)/82 (2.86 SDS score) mmHg Abdominal US: normal, ovaries not visable	Diet advice Metformin 850 mg/12 h
13 years	Obesity: weight 139 kg (+ 5.5 SD), BMI 52, BMI- SDS + 2.93 Final height 164 cm Pubertal status: A2, P4-5, T4, no menarche	Glucose tolerance: HOMA-IR 10.5, HbA1c 5.3 % No dyslipidemia Basal level LH 13.9/FSH 6.2 E/L, stimulated ratio at 60’ 30.3/10.2 E/L, SHBG low (19.3 nmol/l), AMH 1 mcg/L, inhibin B 38.3 ng/L, androgene hormones normal	Bone age advanced + 3SD, growth terminated at 164 cm Hypertension 145(1.88 SDS score)/66 (0.07 SDS score) mmHg Normal bone mineral density CMCRF: hypopnea in sleep, desaturations < 90 %	Diet advice Metformin 2 x 1000 mg In medically supervised weight loss program Noninvasive ventilation during sleep proposed Ramipril 2.5 mg

Blood pressure was elevated already at five years; anti-hypertensive medication was introduced at 13 years of age. Simultaneously, low basal oxygen saturation and hypopnea with marked desaturations during sleep were recorded, and non-invasive respiratory support with continuous positive airway pressure (CPAP) during sleep was proposed.

### Neurodevelopmental History

Gross motor and speech developmental milestones were reached at an appropriate age. On the Verbal Comprehension Wechsler intelligence scale for children (WISC III), performed at the age of 13 years, she showed below-average abilities, especially in understanding common concepts and social situations in the subtests evaluating abstract thinking and on working memory testing. In the first year of life, delayed myelination pattern, with no visible malformations, was determined by magnetic resonance imaging.

### Growth and Puberty

**Figure 3:**
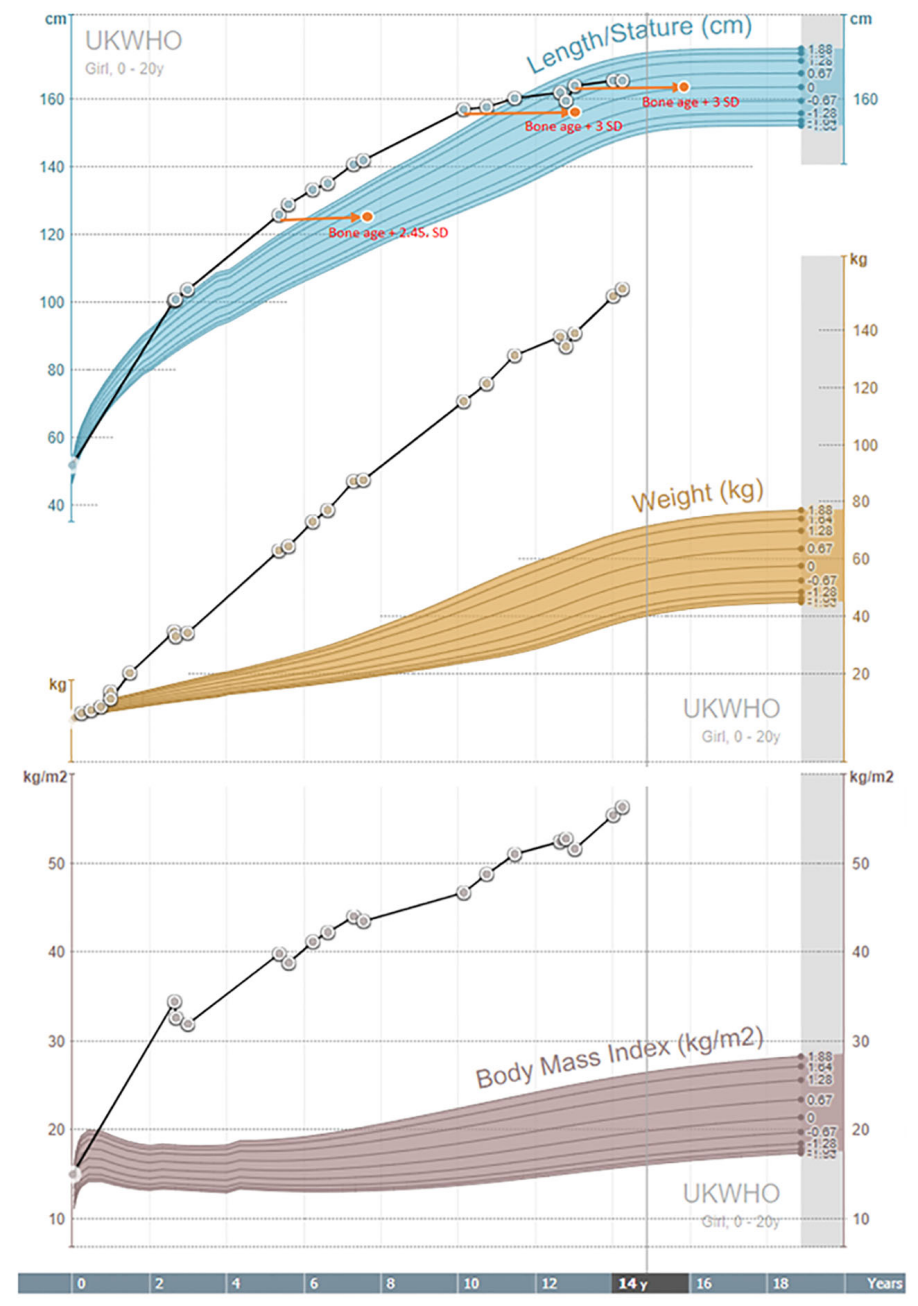
Growth charts.

As illustrated in **Table 1** and **Figure 3**, the girl was tall since the second year of life. Her bone age was advanced since the age of five years. IGF-1 and IGF-BP3 levels were normal (**Table 1**). Adrenarche started at the age of ten years. Aged 13 years, she attained her final height of 164 cm, 2 cm below her target height based on parental heights. At that time, pubertal staging, according to Tanner, was A2, T4, P3, and she did not have menarche. She had LH/FSH levels in the normal range for age and pubertal status.

### Genetic Analysis

Informed consent for genetic testing was obtained from the parents. Genomic DNA was isolated from whole peripheral blood, following standard methods. No mutations in the *ALMS1* gene were found on analysis by PCR and sequencing of both DNA strands of the entire coding region and the highly conserved exon-intron splice junctions.

Next-Generation Sequencing (NGS) was performed using MiSeq desktop sequencer (Illumina, San Diego, USA). The regions of interest were enriched using TruSightOne library enrichment kit (Illumina, San Diego, USA) and targeted analysis of genes causative of cone-rod type of retinal dystrophy (list of genes in the Appendix), with no causative mutations found. The selection of candidate causative variants was extended using PhenIX software (http://compbio.charite.de/PhenIX). 41,5% horizontal coverage of analyzed regions was reached, and 95,7% of target regions of the selected genetic panel were sequenced sufficiently.

In the gene *INPP5E*, a homozygous gene variant c.1897C>T causes a change from glutamine in amino acidic position 633 to stop codon and premature termination of translation (NP_063945.2:p.Gln633Ter). The variant is, to our knowledge, not yet described in The Human Gene Mutation Database, nor present in healthy population studies. Parents, who are consanguineous but healthy, were proved to be carriers of the variant p.Gln633Ter in a heterozygous form. The brother declined all testing.

## Discussion

### Further Phenotypic Characterization of MORMS

MORMS is a very rare autosomal recessive ciliopathy that has been, to our knowledge, so far reported in a single pedigree (13). We contribute the first description of MORMS in a Caucasian pedigree. The most prominent phenotypic features of the presented case were very early retinal dystrophy and early and severe obesity with the development of components of the metabolic syndrome.

Perinatal history of previously described individuals with MORMS (13) and our patient was uneventful; no congenital anomalies were observed. Affected individuals with MORMS in the described Pakistani kindred (13) had poor night vision evident in the first year of life; by the age of three, reduced visual acuity was apparent, but no further visual loss was noticed after that. Unlike in our patient, no nystagmus was evident. In previously described patients ophthalmologic examination of the fundi showed a mildly atrophic retina with thinned blood vessels, without increased pigmentation or optic atrophy. In our patient a complete ophthalmological evaluation was done in the first year of life. The diagnosis of retinal dystrophy was made on the grounds of both clinical and electrophysiological criteria. This is the first description of the retinal dystrophy associated with MORMS being of cone-rode type, similar to other ciliopathies (**Table 2**). Importantly, visual loss in our patient was progressive in the 1^st^ decade of life (**Figure 2**), which should be considered in prognosis and management decisions of other MORMS patients.

**Table 2:** Phenotype comparison between MORM, Bardet-Biedl, Cohen, Alstörm, and Joubert 1 syndrome (OMIM database).

Syndrome	MORM	Bardet-Biedl	Cohen	Alstörm	Joubert 1
Inheritance	AR	AR, Digenic recessive	AR	AR	AR
Gene location/Gene	9q34.3/ INPP5E	1p35.2/ CCDC28B3q11.2/ ARL611q13.2/ BBS1	8q22.2 /VPS13B	2p13.1 /ALMS1	9q34.3 / INPP5E - Variable phenotype - Genetic heterogeneity
Growth/Weight	-Truncal obesity (childhood), *early childhood morbid obesity	-Obesity	- Short stature (GHD) - LBW - Truncal obesity (midchildhood)	-Short stature (GHD) -Advanced bone age -Truncal obesity (childhood)	–
Puberty/Other endocrine features	* Insulin resistance - Male: micropenis *timely pubertal development *hypogonadism with primary amenorea, possible PCOS development *dyslipidemia	*-*Male: Hypogonadism (major), Hypogenitalism	- Delayed puberty	- Insulin resistant diabetes/hyperinsulinemia - Diabetes insipidus - Hypothyroidism/ Multinodular goiter - Hyperuricemia - Dyslipidaemia - Female: Menstrual irregularities - Gynecomastia - Male: Hypergonadotropic hypogonadism	
Eyes	- Retinal dystrophy (congenital, nonprogressive) * progressive in 1^st^ decade - Reduced VA by age 3 years - Cataracts (2^nd^/ 3rd decade)	- Rod-cone dystrophy (by 2^nd^ decade) (major) - Retinitis pigmentosa - Retinal degeneration - Strabismus - Cataracts	- Down slanting palpebral fissures, Almond-shaped eyes - Chorioretinal dystrophy - Myopia - Decreased VA - Optic atrophy	- Cone-rod/pigmentary retinal dystrophy - Photophobia and nystagmus (infancy) - Subcapsular cataracts - Bull's-eye maculopathy - Waxy optic disc pallor - Hyperopia - Central and later peripheral visual loss	- Abnormal, jerky eye movements, impaired saccades - Oculomotor apraxia - Coloboma: optic nerve, chorioretinal - Retinal dysplasia/dystrophy - Epicanthal folds - Ptosis
Central nervous system	- Intellectual disability , moderate (apparent by age 4 years) - Delayed language acquisition *not always present	- Speech disorder/delay - Learning disabilities (major) - Developmental delay - Intellectual disability - Ataxia, poor coordination	- Intellectual disability - Hypotonia - Seizures - Delayed motor milestones - Large corpus callosum - Cerebellar hypoplasia	-Developmental delay	- Delayed psychomotor development, intellectual disability - Ataxia; hypotonia - Occipital myelo/meningocele - Hypoplasia/malformation of the brainstem - 'Molar tooth sign' on MRI - Cerebellar vermis hypoplasia/dysgenesis/agenesis - Deep posterior interpeduncular fossa - Thick, elongated sup. cerebellar peduncles
Other		*Mouth and Teeth* - High arched palate - Dental crowding/hypodontia - Small tooth roots *Hands and Feet* - Polydactyly, usually postaxial (major) - Brachydactyly *GIT* - hepatic fibrosis - Mb Hirschsprung *Kidneys* - anomalies (major) *Miscellaneous* - Presence of 4 major features or 3 major and 2 minor features establishes the diagnosis - Clinical manifestation of some forms of Bardet-Biedl syndrome requires a recessive mutation in 1 of the 6 loci plus an additional mutation in a second locus or triallelic inheritance	*Mouth and Teeth* - High, narrow palate - Open mouth appearance - Prominent upper incisors *Head and Face* - Microcephaly - Short philtrum - Maxillary hypoplasia/ micrognathia - Facial hypotonia - Prominent nasal bridge *Hands and Feet* - Narrow - Mild shortening of metacarpals/metatarsals - Transverse palmar creases *Skeletal* - Mild lumbar lordosis - Mild thoracic scoliosis - Joint hyperextensibility - Cubitus, genu valgus *Skin* - Transverse palmar creases *Hematology* - Leukopenia/Neutropenia *Heart* - Mitral valve prolapse *Miscellaneous* - Cheerful disposition - Increased frequency in the Ashkenazi Jewish population and Finland	*Mouth and Teeth* - Gingivitis - Discolored enamel *Hands and feet* - Pes planus *GIT* - Hepatitis, chronic active - Hepatomegaly/steatosis - Elevated serum transaminases *Kidneys* - Nephritis - failure -Structural anomalies *Skeletal* - Hyperostosis frontalis interna - Kyphosis/scoliosis *Skin and hair* - Acanthosis nigricans - Alopecia *Heart* and vascular - Dilated cardiomyopathy (infancy) - Congestive heart failure - Atherosclerosis - Hypertension *Ears* -Hearing loss, progressive sensorineural - Otitis media *Airways* - Asthma - Recurrent infections	*Mouth* - Triangular-shaped open mouth - Protruding tongue/rhythmic tongue movements - Soft tissue tu.of the tongue *Head and face* - Macrocephaly - Prominent forehead - High, rounded eyebrows - Hemifacial spasms *Hands and feet* - Missing phalanges - Polydactyly - feet, postaxial *GIT* - Hepatic fibrosis *Kidneys* - Renal cysts *Ears* - Low-set/'tilted' ears *Airways* - Neonatal breathing dysregulation - Hyperpnea/tachypnea, episodic - Central apnea *Nose* - Upturned nose - Anteverted nostrils *Behavioral Psychiatric Manifestations* - Hyperactivity - Aggressiveness - Self-mutilation

AR, autosomal recessive; GHD, Growth hormone deficiency; LBW, low birth weight; VA, visual acuity. *Observations from our Case Report.

In the previously described subjects with MORMS, truncal obesity developed by the age of five years, while our patient was obese already at the age of nine months. No acanthosis or insulin resistance was reported in the previously described kindred (13). In our patient, hyperinsulinism was diagnosed at the age of 2.5 years, and impaired glucose tolerance developed at ten years. She developed obesity-related arterial hypertension and obstructive hypopnea in sleep at the age of 13 years.

All previously described individuals with MORMS had delayed language acquisition, while our patient did not. Like all other patients from the described kindred (13), a static mild to moderate intellectual disability with typical motor milestones acquisition and no disturbance of muscular tone were present. There is no description of pubertal development in affected females in Pakistani kindred (13) apart from the information that none have had offspring. Pubertal development started spontaneously and timely in our patient at the age of ten years. At the age of 13 years, while still in the premenarchal phase, puberty was assessed biochemically. Levels of gonadotropins were within the normal levels, with LH/FSH ratio of 3:1, no clinical or biochemical indicators of hyperandrogenism were found. AMH was below average according to her age and pubertal stage (15). Previously described individuals with MORMS (13) had normal growth parameters. Our patient was growing above the parental growth channel in the pre-pubertal phase and had advanced bone age (**Table 1** and **Figure 3**), most probably due to severe childhood obesity and hyperinsulinism (16). However, she reached her final height at 13 years, when closed growth plates were determined, with the final height 2 cm below her target height.

The previously described kindred’s (13) nonsense mutation *INPP5E* gene was located at position 627 (13). A novel nonsense gene variant located in the near vicinity at position 633 was identified in our patient and results in premature termination of mRNA translation and impaired function of the INPP5E protein, as determined by *in silico* methods. Therefore, both pedigrees with MORMS had a truncating gene variant with a similar genetic location in the terminal exon of *INPP5E*, resulting in the omission of terminal amino acids and loss of the C-terminal transmembrane CaaX domain. The p.Gln627Ter variant in the Pakistani pedigree changes the ciliary localization of the affected cells, while the 5-phosphatase activity of the protein is retained (5). The protein variant in our patient was only six amino acids longer; therefore, similar functional consequences could be expected, reflected by similarities in clinical presentation and advocates for a significant correlation between genotype and phenotype.

### Differential Diagnosis of MORMS

Childhood-onset obesity is a consistent feature of monogenic obesity syndromes. It is helpful to categorize obesity syndromes as those with dysmorphisms or developmental delay and those without these features (17). Ciliopathies are clinically and genetically heterogeneous group of monogenic obesity causes, where the common denominator is early-onset obesity in association with retinal dystrophy and developmental delay. The presented patient had moderate developmental delay and retinal dystrophy but no significant dysmorphisms.

Our first clinical differential diagnosis was Alström syndrome (**Table 2**), characterized by cone-rod retinal dystrophy, cardiomyopathy, and type 2 diabetes mellitus (18); however, a mutation of *the ALMS1* gene was not found in our patient.

MORMS is also phenotypically and genetically distinct from the Bardet-Biedl syndrome (BBS; OMIM #209900) and Cohen syndrome (COH1; OMIM # 216550) by the age of onset and progression of the visual impairment, and the lack of several characteristics, including characteristic facies, skin or gingival infection, microcephaly, ‘mottled retina’ and polydactyly (19, 20) (**Table 2**).

Joubert syndrome and related disorders (JBTS) are clinically and genetically heterogeneous ciliopathies that share peculiar midbrain-hindbrain malformation named the ‘molar tooth sign’. JBTS1 shares the affected gene *INPP5E* with MORMS (10). It combines neurological signs with variable multi-organ involvement - the retina, kidneys, liver, and skeleton. The cardinal features are hypotonia that evolves to ataxia and developmental delay, altered respiratory pattern in the neonatal period, and abnormal ocular movements. The development of language skills is typically delayed, also mild to severe intellectual disability is common. Retinal dystrophy has a progressive course with variably conserved vision in JBTS (10). Our patient displayed some clinical characteristics of JBTS: intellectual disability, nystagmus, and progressive retinal dystrophy. Brain magnetic resonance imaging did not show pathological changes; she lacked hypotonia, ataxia, and respiratory pattern disorder and did not present any renal, hepatic, or skeletal involvement to fit the diagnostic criteria (**Table 2**).

MORMS also shares several features with common metabolic syndrome, namely obesity, hyperinsulinemia, and hypertriglyceridemia.

The assessment of severe obesity in children and adults should include screening for potentially treatable endocrine and neurological conditions and genetic diagnosis, also for genetic counseling. Clinical evaluation needs to address complications of severe obesity (21) regardless of the underlying cause.

In conclusion, a Caucasian girl with MORMS due to a novel *INPP5E* truncating variant is presented and compared to the only so far described MORMS pedigree. The main novel clinical finding is a progressive course of cone-rod type retinal dystrophy in the first decade of life, which is essential for prognosis and management decisions. Severe obesity develops in the first year of life, leading to the early development of prediabetes, dyslipidemia, and arterial hypertension. Delay in language acquisition is not necessarily present in association with below-average cognitive abilities.

## Data Availability Statement

The data analyzed in this study is subject to the following licenses/restrictions: Unidentifiable human data. Requests to access these datasets should be directed to primoz.kotnik@mf.uni-lj.si.

## Ethics Statement

The patients’ parents gave their written informed consent for the publication of the report.

## Author Contributions

The corresponding author attests that all listed authors meet authorship criteria and that no others meeting the criteria have been omitted. ADT: Data Curation, Writing – Original Draft and Editing. MAS: Conceptualization, Clinical management of the patient, Data interpretation, Manuscript Editing. SB: Clinical and Genetic diagnostic management. KTP: Genetic analysis and diagnosis confirmation. MD: Genetic analysis, diagnosis confirmation. BSK: Ophthalmological evaluation, diagnostics, and management of the patient, Editing of the Manuscript. TB: Conceptualization, Resources, Supervision. PK: Conceptualization, Writing – Review and Editing, Supervision. All authors contributed to the article and approved the submitted version

## Conflict of Interest

The authors declare that the research was conducted in the absence of any commercial or financial relationships that could be construed as a potential conflict of interest.
