# Supplementary material for: Novel Insights Into Monogenic Obesity Syndrome Due to INPP5E Gene Variant: A Case Report of a Female Patient
Source: Front Endocrinol (Lausanne). 2021 Jun 15;12:581134. doi: 10.3389/fendo.2021.581134 (PMC8241224; doi:10.3389/fendo.2021.581134)
Supplement: Supplementary file 1 [file DataSheet_1.docx]

**APPENDIX**

List of analysed genes causative of cone-rod type of retinal dystrophy.

| ABCA4 | ADAM9 | AIPL1 | BEST1 | C8ORF37 | CABP4 | CACNA1F | CACNA2D4 |
| --- | --- | --- | --- | --- | --- | --- | --- |
| CDHR1 | CERKL | CNGB3 | CNNM4 | CRX | GUCA1A | GUCY2D | KCNV2 |
| PDE6C | PDE6H | PITPNM3 | PROM1 | PRPH2 | RAX2 | RDH5 | RGS9 |
| RGS9BP | RIMS1 | RPGR | RPGRIP1 | SEMA4A | UNC119 | NBL | LART |
| OTX2 |  |  |  |  |  |  |  |
